# Qualitative study on ethics in paediatric Systemic Inflammatory Response Syndrome (SIRS) research: perspectives of Turkish legal guardians

**DOI:** 10.1186/s12910-025-01350-x

**Published:** 2025-11-27

**Authors:** Silviya Aleksandrova-Yankulovska, Paul Thiemicke, Meral Ekşi, Sezgin Şahin, Amra Adrovic, Ozgur Kasapcopur, Marcin Orzechowski, Catharina Schuetz, Florian Steger

**Affiliations:** 1 Institute of the History, Philosophy and Ethics of Medicine, Ulm University, Oberberghof 7, 89081 Ulm, Germany; 2 Cerrahpasa Medical School, Department of Pediatric Rheumatology, Istanbul University-Cerrahpasa, Istanbul, Turkey; 3 Department of Pediatrics, Medizinische Fakultaet Carl Gustav Carus an der Technischen Universitaet Dresden, Dresden, 01307 Germany; 4 German Center for Child and Adolescent Health (DZKJ), partner site Leipzig/Dresden, Dresden, Germany; 5 German Center for Child and Adolescent Health (DZKJ), partner site Ulm, Ulm, Germany

**Keywords:** AI (Artificial intelligence), Group solidarity, Personal autonomy, Research ethics, Systemic inflammatory response syndrome (SIRS)

## Abstract

**Background:**

This qualitative study of the opinions of legal guardians of minors with Systemic Inflammatory Response Syndrome (SIRS) in Turkey identifies and analyses the ethical challenges accompanying the management of paediatric SIRS patients and the collection of genetic data as part of a large multiomics dataset for the development of an AI-based tool for diagnostics and management of SIRS.

**Methods:**

Between January and June 2024, 14 problem-centred, semi-structured exploratory interviews with legal guardians of children with SIRS were conducted in Istanbul, Turkey. The interview consisted of 19 open-ended questions. The interviews were digitally recorded, translated into English, and two independent researchers analysed the content of the information. Qualitative content and thematic analysis were performed to identify major ethical issues.

**Results:**

The analysis identified five major topics: solidarity, autonomy, informed consent, protection of privacy, and AI-driven ethical issues. Solidarity was the most prominent topic and encompassed the aspects of motivation for participation, raising awareness, supporting communication, and loosening data protection. Parents expressed high respect for children’s autonomy. In the vein of the triadic relationship model, the necessity of children with SIRS participating in decision-making was supported by the interviewees as well as the reconfirmation of informed consent in case of future use of collected genetic data. Regarding the development of AI-tool for SIRS, five principles were identified: wide representation, confidentiality, trustworthiness, human control, and orientation.

**Conclusion:**

Our study contributes to the understanding of the ethical challenges accompanying the study of diagnostics using multi-omics data and derived treatment strategies of SIRS in children and adolescents. Different functions of solidarity linked it to the other major topics, thus composing a coherent picture of the ethical aspects of paediatric SIRS research. The identified principles of AI-development correspond to those in the Assessment List for Trustworthy Artificial Intelligence (ALTAI); thus, our research confirmed their real-life relevance and usefulness.

**Supplementary Information:**

The online version contains supplementary material available at 10.1186/s12910-025-01350-x.

## Introduction

Systematic Inflammatory Response Syndrome (SIRS) is a serious acute medical condition characterised by autoinflammation and possible organ failure, caused by a variety of triggers, which include, but are not limited to, an infection [1]. SIRS is often associated with sepsis conditions, thus being of high importance as a cause of death for children worldwide [2]. Recently, criteria distinguishing paediatric sepsis versus SIRS have been redefined [3].

The epidemiology of SIRS is still poorly understood [1, 4] and efforts are being made to apply omics research for improved diagnosis and more targeted treatments [5], including the development of supporting AI-tools [6, 7]. The accompanying ethical challenges are still understudied, but the focus is placed on issues around avoidance of social biases [8], transparency in the development of and equal access to AI-tools, as well as protection of sensitive genetic patients’ data [9]. Children are a widely recognised vulnerable group [10, 11], both for treatment and research. The latter requires meeting preconditions such as specific group benefit, irreplaceability of the research subjects, and minimal risk [12]. Additionally, SIRS context entails emotional stress for the families and complicates the informed consent process [13]. Hence, SIRS treatment decisions often require additional ethics expertise and clinical ethics support. The studies of Civaner et al. (2009), Ekmekçi et al. (2016), and Durmaz et al. (2023), however, point to difficulties with clinical ethics decision-making on different levels due to the lack of ethics committees specialised in consultation services [14, 15], lack of medical ethics post-graduate specialisation [16] and insufficient additional ethics training for paediatricians [14].

Our goal is, based on the specific example of SIRS-research with children and adolescents in one study site in Istanbul, Turkey, to identify and analyse the ethical challenges accompanying the management of paediatric SIRS patients and the collection of genetic data as part of multiomics data for the development of AI-based tool for diagnostics and management of this disease.

## Methods

We conducted 14 problem-centred, semi-structured exploratory interviews with legal guardians of children with SIRS, specifically Familial Mediterranean Fever (FMF), in Cerrahpasa Medical Faculty Hospital in Istanbul, Turkey. In all cases, the legal guardians were the parents of the children. Therefore, we will further use both terms interchangeably. A purposive sampling procedure was applied. We directly contacted the legal guardians of children with SIRS treated in this medical centre and invited them to participate. A written information sheet and oral information with instructions about the topic and course of the interviews, as well as confidentiality and de-identification procedure, were provided to all interested participants. The children whose parents were interviewed ranged in age from 4 to 17 years. A total of 16 parents, 10 mothers and 6 fathers, participated in the interviews. In two of the interviews, both parents took part. The inclusion criteria were: child with inflammatory systemic disease; age range from 29 days to 18 years; stable clinical condition. Excluded were parents of deceased children and those who did not provide informed consent.

The research project was presented to the Research Ethics Board of the Cerrahpasa Medical Faculty Hospital, which decided that, for this interview-based investigation without retrieval of personal information or information about the health or sexuality of the participants, no ethics approval was necessary.

In all interviews, an interview strategy [17], according to an originally developed questionnaire for this study, with 19 open-ended questions (Supplementary Material 1), was employed. The interviews were conducted face-to-face in the period January - June 2024 in the native language of the participants. The interviewer was a paediatric resident from Cerrahpasa Hospital with extensive experience in conducting similar interviews.

The interviews were digitally recorded, transcribed, translated into English, and anonymised. Two independent researchers analysed the content of the information using the methods of qualitative content analysis [18] and thematic analysis [19, 20]. The content was manually coded to identify the main thematic elements and corresponding participants’ statements. Further, the identified statements were extracted from the text body of the transcript and systematised through clustering into topics and subtopics. These mirror important recurring themes touched upon during the interviews. For an illustration of the presented themes, sample quotes were selected.

## Results

**Fig. 1:**
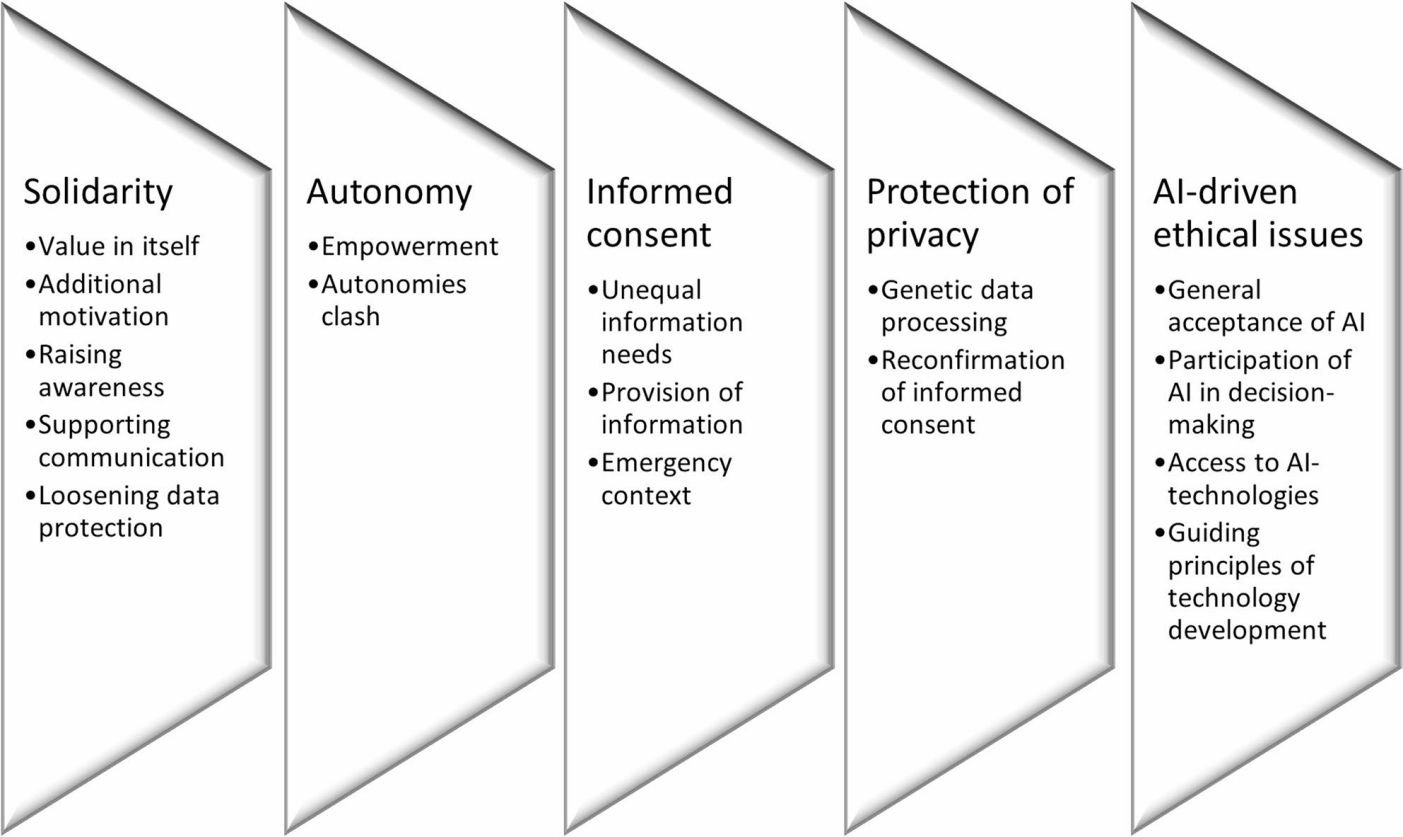
Major topics and respective subtopics identified in the thematic analysis

The analysis identified five major topics: 1) solidarity; 2) autonomy; 3) informed consent; 4) protection of privacy; 5) AI-driven ethical issues (Fig. 1). Among them, solidarity was found to be the main topic in interviewees’ opinions, and will therefore be prioritized in our analysis.

### Solidarity

The context of SIRS and omics research presents a unique combination where participating children do not experience a direct benefit for their own health but can contribute to the accumulation of data that would allow advancement of SIRS diagnostics and therapy for all affected patients. Thus, the future prognosis of other children with SIRS can be improved. This specific meaning of the principle of social justice we labelled as solidarity. The legal guardians nearly unanimously claimed that the main reason for their child’s participation in research was the urge to help.

*“We should not limit our thinking only to the individual. And also other people’s…” (i9)*.

Several interviewees went further in the expression of solidarity, strengthening its understanding as a value in itself.

*“I thought that it would be useful (…) for our children whose treatment was not available*,* or for the children we lost (…) Solidarity is always something nice.” (i5)*.

Other opinions showed empathy with SIRS patients.

*“My daughter also has [the disease] (…) I wanted to participate with the same idea in my mind. Those people are like my children.” (i13)*.

Solidarity also served as additional motivation for participation.

*“I think it [the research] can first be explained in relation to the disease (…) then how other children can benefit from this project.” (i14)*.

Participating in research to “set an example” was another notion of solidarity that we identified. It can be described as raising awareness about certain aspects of SIRS at two levels: increasing the general knowledge about SIRS in the community and the knowledge of the individual child.

*“Because of those who are not aware. So that they might be informed (…) To set an example (…) for their other friends*,* I believe it is beneficial for them to behave in a way that is sensitive to their community (…) It’s good for the next generation’s future.” (i7)*.

*“She should participate so that she can be informed (…) when this child grows up and gets married*,* she can remember it. Maybe*,* her child might develop the same condition.” (i11)*.

Further on, solidarity was found to support communication.

*“You are going through this problem*,* your other friends may also go through similar problems*,* and would you accept this research in order to help them?” (i9)*.

Generally, parents were not concerned about genetic data collection, storage and processing, because they saw a potential benefit for other affected children. In that way, solidarity leads to a kind of “loosening” of data protection.

*“If it’s going to help the kids*,* I’m not concerned [about the genetic data collected] in any way.” (i14)*.

### Autonomy

The empowerment of children was considered important by all interviewees. This meant the provision of understandable information to children to enable their participation in the decision-making process.

*“Children*,* nowadays*,* actually understand certain things. They are already aware of the situation because they have experienced the disease process themselves. When we tell them what kind of a journey this disease is and that it will be useful in helping other children*,* I think they will actually appreciate it.” (i10)*.

Our interviewees perceived that their children’s gradual development led to an increasing ability to understand. The disease experience itself may contribute to a child’s maturation.

*“Since she is on medication (…) you can teach her somehow*,* she can understand (…) you may not be able to explain it to a healthy*,* not sick child*,* but you can explain it to a child who has a disease.” (i12)*.

Parents demonstrated a very favourable attitude towards the use of visual aids in the information process.

*“I think it would be better and more appropriate to explain this to a 5-year-old child by showing pictures*,* or to a 10-year-old child by visuals/videos*,* and to children after the age of seventeen or fifteen by giving information to each other in the same way we do now [without visual aids].” (i1)*.

An exceptional real-life example that deserves special attention was shared by one interviewee: their child, who was an inpatient, went as far as informing other hospitalised children about the treatment procedures.

*“… in the phlebotomy room*,* when there are a few children*,* he says*,* “Mom*,* wait a minute. Can I say something to my friend?” We don’t know him/her at all (…) The 6-year-old goes to 8–10-year-olds (…) and tries to explain this.” (i5)*.

When it comes to the parents’ view about the possible collision of children’s and parents’ opinions, the results varied. On the one side were the opinions of legal guardians who considered that “parents’ decisions matter more” (i4), whereas children can “get involved together with the family” (i1) and depending on “to what period they are in” (i2, i14) and “if they are of sound mind” (i5). On the other side were the opinions that the child’s involvement was indisputable because “the child lives his/her own life” (i3) and “[disease] is something that happened to him/her” (i9).

Whenever parents’ and children’s autonomies clashed, the spectrum of opinions unfolded similarly: on the one side, the opinion of the parents was seen as more important, while other parents placed the child’s opinion at the centre. Some interviewees provided additional arguments to explain their views. The child’s opinion mattered more because “it is her right” (i11), “in his mind he was already thinking about his own disease” (i5), while “parents don’t experience this problem” (i9). Parents’ opinion, on the other side, was considered more important because “children may not be able to think in that regard, they may not see it like we do.” (i12).

### Informed consent

The informed consent process was discussed from many perspectives. First of all, bearing in mind the complexity of information about SIRS and omics research, we investigated the perceptions about the provision of information. Most of the interviewees seemed to have no particular difficulties with understanding the information. Trusting their physician and the simpler content of the information were pointed out as important.

Children and parents were considered to have unequal information needs.

*“I think it is more important to inform the parents more extensively than the child. Because the parents take care of that child and I think it is more important that the parents are better informed about what the parents should do when the child experiences an attack or that the parents should know the solutions better.” (i1)*.

Inevitably, the information process was influenced by the emergency context of SIRS. The parents shared that they had difficulties coming to a decision. They “didn’t know what to do” (i13), it was “difficult to make decision at that moment” (i9), they “run hot and cold” (i3), and “trusted the doctors” (i3) or relied on their own experience with the disease.

*“When something happens to our children*,* in that moment*,* that is*,* in terms of being able to stay calm. Because I went through the same thing. (…) I usually tried to stay calm because I knew it from myself.” (i7).*

### Protection of privacy

Omics research which our research on SIRS is based on, involves genetic data processing. Generally, parents were not concerned about it. Several conditions were set though, i.e., trust in physicians, safeguarding privacy, proper informed consent, good purpose, and non-invasiveness in the obtainment of samples.

*“There is no problem*,* if we are informed when they are going to be taken*,* not without our knowledge*,* but rather when the information is given and then the results are shared with us. But if they are taken as guinea pigs without my knowledge and their genetic findings are used or analyzed without any information being given about it*,* it would inevitably disturb everyone.” (i1)*.

Concerning the future usage of genetic data, many parents envisaged reconfirmation of the informed consent either by them or by the child him/herself at a later time point.

*“I can decide now*,* then maybe she can decide when she becomes an adult…Yes*,* I think she should be consulted.” (i12)*.

### AI-driven ethical issues

Of special interest were ethical issues related to the development of AI-tool for diagnosis and treatment of SIRS. In our study, the acceptance of AI showed variations with both negative and positive views, while the opinions towards AI participation in decision-making were remarkably unanimous. Almost all interviewees pleaded for physicians’ decision-making, underlying the importance of face-to-face communication and discussion. AI decision-making, on the other side, was associated with the risk of misjudgment and the impossibility of perceiving the reaction of the other person.

*“A doctor can feel something by a touch*,* or (…) they can understand what disease a child has (…) I don’t know on which grounds artificial intelligence is capable of doing this.” (i3)*.

*“When you talk to a person*,* you can at least see from their eyes what they mean.” (i8)*.

Nevertheless, future benefits of AI decision-making were also stated.

*“The decision that an artificial intelligence (…) can make cannot be compared to the decision that any expert can make when that day comes. Think of it like this: 100 doctors come together and consult for a patient. AI will be able to do much more than that.” (i6)*.

As for the access to the AI-technologies, some interviewees thought that any AI diagnostic tool developed through research should be accessible. The opinions differed only on the scope of accessibility: universal access or access only for specialists or access for patients’ families and physicians.

*“Of course*,* it should be universal. Not just Turkey*,* this country*,* that country. Human beings are always human beings. (…) We can’t discriminate between people*,* religion*,* race.” (i5)*.

When asked what advice they would give to the AI-technology developers, parents pointed to:

1. Wide representation: *“We would like them to test more people in a wider region on the same disease (…) rather than just looking at one person or examining ten people and to come up with a solution.” (i1)*.
2. Confidentiality: *“I want them to value confidentiality. I don’t want that data to be made publicly available. I would like that data to be collected with respect.” (i10)*.
3. Trustworthiness: *“I would like to know how true it is*,* is it really done meticulously or is it just (…) sugar-coated*,* but I want to know if it is just a showpiece or not.” (i4)*.
4. Human control: *“If artificial intelligence advances further at this pace*,* it can be advantageous. Of course*,* if its control remains in the hands of the people.” (i10)*.
5. Human orientation: *“We approve of any human-oriented work that is helpful for people everywhere. As long as it is human-oriented.” (i7)*.

## Discussion

### Solidarity

**Fig. 2:**
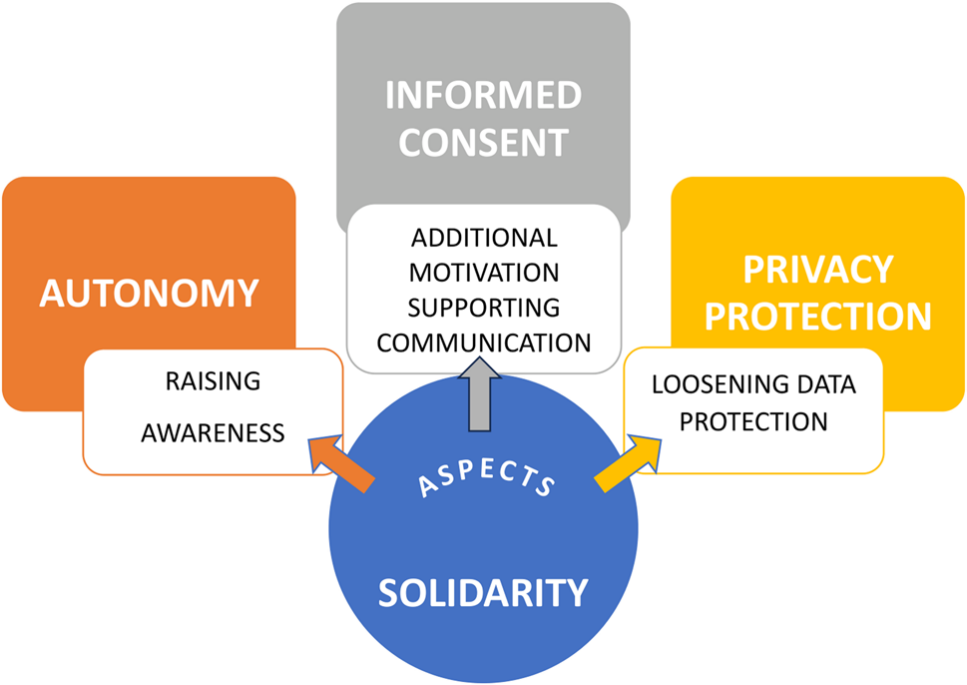
Interrelationship between solidarity as a key identified topic and the other major topics

The genuine interest in the well-being of others [21] serves as a source of motivation for participation in research but also as a value in itself, something inherently positive, defined by compassion towards the fate of others. It is usually observed in close relational networks and is more unlikely to be directed at “anonymous strangers” [22]. However, almost all of our interviewees expressed a strong solidarity towards other patients. One possible explanation, that was identified, could be the empathy towards individuals who also suffer from SIRS. Similar findings were reported for other contexts involving non-autonomous, legally supervised individuals. The most common motivation for research participation was helping other patients with the same condition [23, 24]. Thus, the shared experience may fuel solidarity, despite the lack of a close relation, and plays the role of additional motivation for consenting to participation in research. Consequently, solidarity relates to the topic of informed consent (Fig. 2). Several interviewees mentioned setting an example to others as an important part of their altruistic motivation, and therefore a function of solidarity. In fact, we here observe an intergenerational type of solidarity [25] oriented towards the future, i.e. the child would be better prepared to deal with the same illness if their own children were affected. At that point, solidarity in its function of raising awareness relates to the major topic of autonomy (Fig. 2). Further intertwining of solidarity with autonomy and informed consent was found in its role of supporting communication, where research contributed to other children’s understanding of disease and empowered their decision-making. Additionally, solidarity was connected to the subtopic of genetic data processing where it justified loosening the protection of sensible data. To sum up, we have identified several functions of solidarity, i.e. raising awareness, additional motivation for participation in research, supporting communication, and loosening data protection. Each of these functions linked solidarity to one other major topic, thus composing a coherent picture of the ethical aspects of paediatric SIRS research (Fig. 2). We further discuss results through the principle of autonomy.

### Autonomy

Traditionally, parents and physicians have made medical decisions on behalf of children [26] and in the way they considered to be in children’s best interest. However, new “triadic” models of relationships have developed [27] where children are involved in an age-appropriate manner [28]. The triadic model respects the right of children to be heard regarding any decision affecting their lives [29], but there are variations by country, culture, and even healthcare facility [14]. In Turkey, the majority begins at the age of 18, according to Article 11 of the Turkish Civil Code. Additionally, Article 70 of Turkish Law No. 1219 [30] states that medical decisions concerning children shall be made by their legal representatives. Such wording mirrors Article 6 of the Oviedo Convention [31]. Considering this legal background, our interviewees demonstrated an unusually high respect for their child’s autonomy. This might be due to the severity of the disease and its prolonged course, which made children understand their exceptional circumstances and consequently reach rational decisions before their legal age of maturity [32]. Different interactive tools are proven to be useful in communication with children [33] to facilitate their understanding and empower them to participate in decision-making. Special attention was drawn to situations where parents’ and children’s autonomies clash. These situations can lead to invalid informed consent [34] because in clinical trials the will and the motives of the child should be respected and taken into account. Every effort should be made to reach a consensus, but if this is not achievable, the dissent of either party is decisive. This is even more the case in research with no direct benefit to the child [35], which is the case in our research context. Other challenges of informed consent are related to privacy protection.

### Informed consent and privacy protection

An appropriate informed consent process should be ensured in regard to providing detailed enough information on the research project [28]. The effects of the emergency context on parents’ understanding of information and its subsequent transfer to the child should be considered [13, 36]. An additional complicating factor in the informed consent process is the need to address particularities of genetic data processing [37] and to respect the General Data Protection Regulation (GDPR) [38]. Parents considered reconfirmation of their informed consent necessary in case of any future use of genetic data. This result supported the high respect for child’s autonomy.

As our project was related to the development of AI-prediction model for SIRS, we studied the opinions on access to AI-technologies and related ethical issues.

### AI-driven ethical issues

The equity of access is a classical representation of the principle of justice [39]. At the background of the predominant solidarity theme, it was not surprising to find high support for wide access to AI-diagnostic tools. Also, without being experts in AI-technology assessment, parents in our study were able to identify five guiding principles of technology development that correspond in meaning to the requirements set by the Independent High-level European Commission Expert group in the Assessment List for Trustworthy Artificial Intelligence (ALTAI) [40] – Table 1. The ALTAI document serves as an official guide to AI-developers for self-evaluation of compliance with ethical principles. Accordingly, our research confirmed the real-life relevance of ALTAI requirements. It also underlined the most prominent users’ perspectives that need to be taken into account in the development of an AI-diagnostic tool for SIRS. Most important were the benefits of AI-tools for humans and the ability of human control. The quality of AI-technology, on the other hand, was judged by the completeness of primary databases and trustworthiness.

**Table 1:** Comparison between guiding principles for AI-technology development in our study and ALTAI requirements

Principles defined in our study	Corresponding requirement in ALTAI*
Wide representation	Diversity, Non-discrimination and Fairness
*Meaning: AI to be based on tests of more people in a wider region.*	*Meaning: AI should be user-centric and designed in a way that allows all people to use AI products and services*,* regardless of their age*,* gender*,* abilities or characteristics.*
Confidentiality	Privacy and Data Governance
*Meaning: Confidentiality to be valued in AI-construction so that data are not made publicly available.*	*Meaning: Adequate data governance that covers the quality and integrity of the data used.*
Trustworthiness	Technical Robustness and Safety
*Meaning: How true AI is and whether it has been done meticulously.*	*Meaning: AI ability to deliver services that can justifiably be trusted.*
Human control	Human agency and oversight
*Meaning: AI control should remain in the hands of people.*	*Meaning: Provision of governance mechanisms during the AI development.*
Human orientation	Human agency and oversight
*Meaning: AI should be helpful to people*,* in particular*,* to be used in medicine.*	*Meaning: AI systems should support user’s agency and uphold fundamental rights.*

* (European Commission, 2020)

### Best interest of the child

Lastly, as our research focuses on a severe childhood condition, we wish to pay special attention to the perspective of the best interests of the child. As stated in Article 3(1) of the United Nations Convention on the Rights of the Child (UNCRC) [29], “In all actions concerning children, … the best interests of the child shall be a primary consideration.” Our results, particularly regarding solidarity considerations, clearly demonstrate the priority that parents place on children’s interests. Since the children participated in the project without receiving direct benefits, their involvement - motivated by feelings of solidarity - also contributed to enhancing the best interests of other children suffering from SIRS. Furthermore, Article 12 of the UNCRC stipulates that a child’s own views should be taken into account [29], which aligns with our findings on the empowerment of children through participation in decision-making.

### Limitations

The results of our research are limited by several conditions. The qualitative methodology does not aim at the representativeness of opinions. It has been chosen because of the novelty of the study topic and the acuteness and severity of SIRS as an opportunity to gain deep insight into this research topic. Additionally, we interviewed legal guardians in a single centre in the capital of Turkey. Therefore, our results do not allow for generalisation cross-culturally, let alone from a global perspective.

## Conclusions

In conclusion, our study contributes to the understanding of the ethical challenges accompanying the study of diagnostics using multi-omics data and derived treatment strategies of SIRS in children and adolescents. First, it provides new insights into approaches for empowering children’s decision-making. Second, our study delivers the user’s perspectives that can support the development of AI-tools and their respective guidelines for SIRS patients. Third, in the context of our study, we identified a highly developed and multifaceted sense of solidarity that is worth investigating in other settings, possibly at a more overarching level. In the long run, the preparation of ethics guidelines for SIRS paediatric patients would be justified to support the work of frontline physicians and pave the way for further improvement of care for SIRS patients.

## Supplementary Information

Supplementary Material 1.

## Data Availability

The datasets analysed during the current study are not publicly available due to the sensitivity of the data involved but are available from the corresponding author on reasonable request.

## Abbreviations

ALTAI: Assessment List for Trustworthy Artificial Intelligence
FMF: Familial Mediterranean Fever
GDPR: General Data Protection Regulation
SIRS: Systemic Inflammatory Response Syndrome
UNCRC: United Nations Convention on the Rights of the Child

## References

[1] Foo C, Seabrook J, Sangha G, Foster J. Presumed systemic inflammatory response syndrome in the pediatric emergency department. Pediatr Emerg Care. 2019;35(8):522–6. 10.1097/PEC.0000000000001425. 29438125 10.1097/PEC.0000000000001425

[2] Kannikeswaran N, Mahajan P. Pediatric sepsis: new strategies for reducing sepsis related mortality. Indian Pediatr. 2023;60(12):981–4. 10.1007/s13312-023-3059-y. 37700586

[3] Schlapbach LJ, Watson RS, Sorce LR, Argent AC, Menon K, Hall MW, et al. International consensus criteria for pediatric sepsis and septic shock. JAMA. 2024;331(8):665–74. 10.1001/jama.2024.0179. 38245889 10.1001/jama.2024.0179 PMC10900966

[4] Horeczko T, Green JP. Emergency department presentation of the pediatric systemic inflammatory response syndrome. Pediatr Emerg Care. 2013;29(11):1153–8. 10.1097/PEC.0b013e3182a9e629. 24168885 10.1097/PEC.0b013e3182a9e629

[5] Ruiz-Rodriguez J, Plata-Menchaca E, Chiscano-Camón L, Ruiz-Sanmartin A, Pérez-Carrasco M, Palmada C, et al. Precision medicine in sepsis and septic shock: from omics to clinical tools. World J Crit Care Med. 2022;11(1):1–21. 10.5492/wjccm.v11.i1.1. 35433311 10.5492/wjccm.v11.i1.1 PMC8788206

[6] Koker O, Sahin S, Yildiz M, Adrovic A, Kasapcopur O. The emerging paradigm in pediatric rheumatology: harnessing the power of artificial intelligence. Rheumatol Int. 2024;44(11):2315–25. 10.1007/s00296-024-05661-x. 39012357 10.1007/s00296-024-05661-x PMC11424736

[7] Can Demirbaş K, Yıldız M, Saygılı S, Canpolat N, Kasapçopur Ö. Artificial intelligence in pediatrics: learning to walk together. Turk Arch Pediatr. 2024;59(2):121–30. 10.5152/TurkArchPediatr.2024.24002. 38454219 10.5152/TurkArchPediatr.2024.24002 PMC11059951

[8] Kasapçopur Ö. Anti-racist pediatric research against discrimination in science with diversity, equity, and inclusion. Turk Arch Pediatr. 2022;57(2):116–7. 10.5152/TurkArchPediatr.2022.180222. 35383005 10.5152/TurkArchPediatr.2022.180222 PMC9113388

[9] Boch S, Sezgin E, Lin Linwood S. Ethical artificial intelligence in paediatrics. The Lancet Child & Adolescent Health. 2022;6(12):833–5. 10.1016/S2352-4642(22)00243-7. 36084667 10.1016/S2352-4642(22)00243-7

[10] Kasapçopur Ö. Poverty and discrimination: big enemies of children all over the world. Turk Arch Pediatr. 2023;58(6):564–5. 10.5152/TurkArchPediatr.2023.233110. 37915269 10.5152/TurkArchPediatr.2023.233110 PMC10724717

[11] Patel T. The Ethics of Pediatric Clinical Trials. Intersect: The Stanford Journal of Science, Technology, and Society. 2024;17(2). https://ojs.stanford.edu/ojs/index.php/intersect/article/view/2732/1673. Accessed 7 Feb 2025.

[12] World Medical Association. World Medical Association Declaration of Helsinki: ethical principles for medical research involving human subjects. 2013. https://www.wma.net/policies-post/wma-declaration-of-helsinki-ethical-principles-for-medical-research-involving-human-subjects/. Accessed 7 Feb 2025. 10.1001/jama.2013.281053 24141714

[13] Neuman G, Shavit I, Matsui D, Koren G. Ethics of research in pediatric emergency medicine. Paediatr Drugs. 2015;17(1):69–76. 10.1007/s40272-014-0110-4. 25475848 10.1007/s40272-014-0110-4

[14] Durmaz N, Ulukol B, Şahinoğlu S. Perceptions of pediatric residents and pediatricians about ethical dilemmas: the case of Turkey. Arch Pediatr. 2023;30(8):537–43. 10.1016/j.arcped.2023.06.012. 37714736 10.1016/j.arcped.2023.06.012

[15] Civaner M, Sarikaya O, Balcioğlu H. Uzmanlik eğitiminde tip etiği [Medical ethics in residency training]. Anadolu Kardiyol Derg. 2009;9(2):132–8. 19357056

[16] Ekmekçi P. Medical ethics education in Turkey; state of play and challenges. Int Online J Educ Teach. 2016;3(1):54–63. 27213100 PMC4871155

[17] Braun V, Clarke V. Successful qualitative research: A practical guide for beginners. London: SAGE Publications Ltd; 2013.

[18] Mayring P. Einführung in die qualitative Sozialforschung. 6th ed. Weinheim, Basel: Beltz; 2016.

[19] Braun V, Clarke V. Using thematic analysis in psychology. Qual Res Psychol. 2006;3(2):77–101. 10.1191/1478088706qp063oa.

[20] Vaismoradi M, Turunen H, Bondas T. Content analysis and thematic analysis: implications for conducting a qualitative descriptive study. Nurs Health Sci. 2013;15(3):398–405. 10.1111/nhs.12048. 23480423 10.1111/nhs.12048

[21] Lyons B. Solidarity, children and research. Bioethics. 2012;26(7):369–75. 10.1111/j.1467-8519.2012.01988.x. 22827318 10.1111/j.1467-8519.2012.01988.x

[22] Hoedemaekers R, Gordijn B, Pijnenburg M. Solidarity and justice as guiding principles in genomic research. Bioethics. 2007;21(6):342–50. 10.1111/j.1467-8519.2007.00562.x. 17845458 10.1111/j.1467-8519.2007.00562.x

[23] Brune C, Stentzel U, Hoffmann W, van den Berg N. Attitudes of legal guardians and legally supervised persons with and without previous research experience towards participation in research projects: a quantitative cross-sectional study. PLoS One. 2021;16(9):e0256689. 10.1371/journal.pone.0256689. 34525101 10.1371/journal.pone.0256689 PMC8443074

[24] Hens K, Nys H, Cassiman J, Dierickx K. Risks, benefits, solidarity: a framework for the participation of children in genetic biobank research. J Pediatr. 2011;158(5):842–8. 10.1016/j.jpeds.2010.12.036. 21349539 10.1016/j.jpeds.2010.12.036

[25] Ellerich-Groppe N, Pfaller L, Schweda M. Young for old—old for young? Ethical perspectives on intergenerational solidarity and responsibility in public discourses on COVID-19. Eur J Ageing. 2021;18(2):159–71. 10.1007/s10433-021-00623-9. 33967661 10.1007/s10433-021-00623-9 PMC8093129

[26] Dickenson D. Children’s informed consent to tretment: is the law an ass? J Med Ethics. 1994;20(4):205–6. 10.1136/jme.20.4.205. 7861423 10.1136/jme.20.4.205 PMC1376555

[27] Harrison C, Kenny N, Sidarous M, Rowell M. Bioethics for clinicians: 9. involving children in medical decisions. CMAJ. 1997;156(6):825–8. 9084389 PMC1227047

[28] Roth-Cline M, Nelson R. Parental permission and child assent in research on children. Yale J Biol Med. 2013;86(3):291–301. 24058304 PMC3767214

[29] United Nations General Assembly. Convention on the rights of the child. 1989. https://www.ohchr.org/en/instruments-mechanisms/instruments/convention-rights-child. Accessed 7 Feb 2025.

[30] Gazete R. Tababet ve Suabat ¸ ı San’atlarının Tarzı _ Icrasına Dair Kanun. 2008. https://www.mevzuat.gov.tr/mevzuatmetin/1.3.1219.pdf. Accessed 7 Feb 2025.

[31] Council of Europe. Convention for the protection of Human Rights and Dignity of the Human Being with regard to the Application of Biology and Medicine: Convention on Human Rights and Biomedicine (ETS No. 164). 1997. https://www.coe.int/en/web/conventions/full-list?module=treaty-detail&treatynum=164. Accessed 7 Feb 2025.

[32] Carey S. Conceptual change in childhood. Cambridge: The MIT Pres; 1987. p. 9780262530736.

[33] Cantrell K, Patel N, Mandrell B, Grissom S. Pediatric HIV disclosure: a process-oriented framework. AIDS Educ Prev. 2013;25(4):302–14. 10.1521/aeap.2013.25.4.302. 23837808 10.1521/aeap.2013.25.4.302

[34] Expert group on clinical trials for the implementation of Regulation (EU) No536/2014 on clinical trials on medicinal products for human use. Ethical considerations for clinical trials on medicinal products conducted with minors. 2017. https://health.ec.europa.eu/system/files/2018-02/2017_09_18_ethical_consid_ct_with_minors_0.pdf. Accessed 7 Feb 2025.

[35] Waligora M, Różyńska J, Piasecki J. Child’s objection to non-beneficial research: capacity and distress based models. Med Health Care Philos. 2016;19(1):65–70. 10.1007/s11019-015-9643-8. 25916607 10.1007/s11019-015-9643-8 PMC4805702

[36] Doyle R, McBride C, Forster E, Petsky H. Insisting on prospective consent in paediatric critical care research may be throwing the baby out with the bathwater. J Paediatr Child Health. 2022;58(9):1520–4. 10.1111/jpc.16144. 35932459 10.1111/jpc.16144 PMC9545375

[37] Shabani M, Borry P. Rules for processing genetic data for research purposes in view of the new EU General Data Protection Regulation. Eur J Hum Genet. 2018;26(2):149–56. 10.1038/s41431-017-0045-7. 29187736 10.1038/s41431-017-0045-7 PMC5838983

[38] European Parliament and the Council of the European Union. Regulation (EU) 2016/679 of the European Parliament and of the Council of 27 April 2016 on the protection of natural persons with regard to the processing of personal data and on the free movement of such data (GDPR). 2016. https://eur-lex.europa.eu/eli/reg/2016/679/oj. Accessed 7 Feb 2025.

[39] Beachamp T, Childress J. Principles of Biomedical Ethics. 8th ed. Oxford: Oxford University Press; 2019. https://doi.org/9780190640873.

[40] European Commission. The assessment list for trustworthy artificial intelligence (ALTAI) for self assessment. Brussels: EU; 2020. 10.2759/002360.

