# Supplementary material for: Qualitative study on ethics in paediatric Systemic Inflammatory Response Syndrome (SIRS) research: perspectives of Turkish legal guardians
Source: BMC Med Ethics. 2025 Nov 27;26:170. doi: 10.1186/s12910-025-01350-x (PMC12690800; doi:10.1186/s12910-025-01350-x)
Supplement: Supplementary file 1 — Supplementary Material 1. [file 12910_2025_1350_MOESM1_ESM.docx]

| **TIPS – INTERVIEW GUIDE: Parents and legal guardians** | | |
| --- | --- | --- |
| **Topics and questions** | | **Content-related focus** |
| **Project Information and consent**  SIRS is a sudden and uncontrolled overreaction of the body’s own immune system. This is a life-threatening condition. The body’s reactions in SIRS are very non-specific, making it very difficult for doctors to decide on a specific treatment. Diagnostic tools that analyse individual genetic data and distinguish between infectious and non-infectious causes of disease would improve patients’ outcome.  Therefore, the aim of the TIPS study, in which you participate, is to identify diagnostic patterns to enable individual-tailored therapies for pediatric patients.  Analysis of genetic data from pediatric patients raises specific ethical concern, related most to the fact that the participants will not directly benefit from the developed new diagnostic and treatment predictive tool. Thus, careful consideration must be given to information process, individual risks and benefits, and processing of patients’ data. These ethical challenges will be analysed by the Ulm team as part of TIPS consortium.  For fulfilling this task we use a qualitative approach by interview. The interview will last approximately 60 minutes and will take place digitally via Zoom, hosted at Ulm University. Secure end-to-end encryption will be applied. The interview will be digitally recorded, stored on Ulm University’s secure servers, and then transcribed. Until the transcription is complete, it is possible to delete the interview in case of withdrawal. After transcription, the digitally recorded interviews will be deleted. Participation in the interview is voluntary and will in no way affect care to the patients. All data will be treated confidentially. Recording and further processing of all data will be pseudonymised. Pseudonymised means that your personal data are removed and substituted by codes so that you cannot be directly identified. To ensure confidentiality, any details mentioned during the interview that could provide information about the interviewees’ identity will be deleted. Only the researchers in this team will have access to the recordings and transcripts.  The results will be published in scientific articles and publicly presented in fully anonymised form. This means that no names or other information will be mentioned that would allow to identify you. | | **General information for the disease**  **Aim**  **Specific task of Ulm University team**  **as part of TIPS consortium**  **Interview procedure**  **Data processing**  **Results utilisation** |
| **Thematic introduction**  The project involves research on children with SIRS, which is a life-threatening, rare disease with unknown origins. Additionally, different children respond differently to “standard” treatment. That’s why the scientists assumed that certain biological characteristics, like for example genes, can determine the best therapy for each child. To understand these characteristics physicians-researchers will collect biological materials like blood, cerebrospinal fluid, urine and stool. Through analysis of all data they aim to create predictive computerised tool to find the best therapy for each child with SIRS in the future. To do that in the best way they need clinical data from many children with different characteristics.  In our interview, we would like to ask you about your experience related to your participation in the project, about your possible concerns regarding the procedure and about possibility for improvement of similar projects in the future, especially when it comes to children participation. | | **OMICS meaning**  **AI**  **Datasets**  **Introduction to the questions** |
| **Questions** | **Optional questions** |  |
| **Introductory questions** | | |
| 1. Why did you decide for your child to participate in the project? |  | **Attitude and expectations** |
| 2. Do you think that it is good that children are included in research? |  | **General stand** |
| **Topic: Autonomy** | | |
| 3. Should children be involved in the decision process (in research)? | *Why should children be involved in the decision??* | **Involvement in research of people who are not autonomous to decide for themselves** |
| 4. How can children be enabled to understand what they will be involved in? |  | **Empowerment of decision-making** |
| 5. What if the child opinion differs from yours? Which opinion should weight more? |  | **Stand on potential disagreement** |
| **Topic: Informed consent and process of information** | | |
| 6. Did you find the information about the project complex and difficult to understand? | *Was it more complex to understand than usual?* | **Understanding of information** |
| 7. Do you think that you and your child should be informed separately? |  | **Information process** |
| 8. What do you think about having more than one information meetings? |  |  |
| 9. What do you think about using aids like pictures, videos, colouring books when informing children? | *What (else) could improve your information and your child’s information?* | **Form of information process** |
| 10. Has your child ever been in emergency situation before? How difficult was it for you to decide for your child now? | *Why it was (was not) difficult?* | **Surrogate decision-making in critical situation** |
| **Topic: Risk and benefits of the research** | | |
| 11. Should children participate in research that does not has direct individual benefit for them? | *In your opinion, what is the highest acceptable risk in that case?* | **Non-therapeutic research on children** |
| **Topic: Protection of privacy** | | |
| 12. Are you concerned about the genetic data (hereditary information) being collected from your child? | *What are your concerns?* | **Additional protection of genetic data** |
| 13. Are you concerned that the genetic data collected from your child can be stored for many years and used in other research? | *What are your concerns?* | **Biobanking** |
| 14. If the data is used in future other research, do you think that you or your grown-up child should be approached again for consent? Or do you think that one decision is enough? | *Why do you think that you or your child should be recontacted?* | **Future usage of biodata** |
| **Topic: Social justice in relation to Artificial Intelligence use** | | |
| 15. One of the results of this project could be an Artificial intelligence tool for diagnosis and treatment of SIRS. How should the access to it be arranged? |  | **Equal access to new technologies** |
| 16. Do you have concerns that in the future computers can decide about treatment of children? | *What are your concerns*? | **Use of AI in the medicine** |
| 17. Would you prefer that decisions for your child would be always taken by a doctor? | *Why?* | **Trust in decisions made by AI** |
| **Topic: Solution approaches** | | |
| 18. What would you advice the developers in this project to think of when they work on the artificial intelligence tool? |  | **Values in AI development in SIRS** |
| 19. Is there anything that could improve your experience with the involvement in this project? |  | **General impression of research in emergency situation** |
| **Closing remarks and information**  Thank you for your participation in this interview. Is there anything else that you would like to add? | |  |
